# Chronic Jet Lag Exacerbates Jejunal and Colonic Microenvironment in Mice

**DOI:** 10.3389/fcimb.2021.648175

**Published:** 2021-06-01

**Authors:** Qing Li, Bo Wang, Hong-Yi Qiu, Xiu-Juan Yan, Li Cheng, Qian-Qian Wang, Sheng-Liang Chen

**Affiliations:** Division of Gastroenterology and Hepatology, Renji Hospital, Shanghai Institute of Digestive Disease, School of Medicine, Shanghai Jiao Tong University, Shanghai, China

**Keywords:** colon, jejunum, jet lag, metabolites, microbiota

## Abstract

**Background:**

Evidence suggests that circadian rhythm disorder is associated with a variety of gastrointestinal diseases, and the circadian rhythm plays a key role in maintaining the homeostasis of intestinal flora. The underlying mechanisms are still not completely identified. This study was aimed to explore whether jet lag-caused circadian disruption influences gut microbiome and its metabolites.

**Methods:**

Mice were synchronized with 12-h light/dark cycles (control group) or subjected to daily 8-h advance of the light/dark cycle for every 3 days (jet-lagged group). Four months later, fecal samples and jejunal contents were collected and analyzed by 16S rRNA gene sequencing. In addition, fecal samples were subjected to metabolome analysis with ultra-performance liquid chromatography coupled to tandem mass spectrometry (UPLC-MS/MS).

**Results:**

The results of 16s rRNA sequencing showed that chronic jet lag led to decreased microbial abundance, richness, and diversity in both feces and jejunal contents. ANOSIM analysis revealed significant difference between control and jet-lagged groups. As the colonic microbiome, the abundance of Bacteroidetes phylum was significantly decreased and that of Actinobacteria phylum was increased in jet-lagged mice. Jet lag increased the ratio of Firmicutes to Bacteroidetes, an indicator for the imbalance of gut microbiota. Metabolome analysis of fecal samples showed that the levels of tryptophan and its derivatives were decreased in jet-lagged mice. In addition, fecal levels of secondary bile acids changed under jet lag conditions. Correlation analysis identified associations between tryptophan (and its derivatives) levels and colonic microbiota.

**Conclusions:**

This study presents a comprehensive landscape of gut microbiota and its metabolites in mice subjected to chronic jet lag. The results suggest that circadian disruption may lead to changes in fecal and jejunal microbiota and fecal metabolites. Moreover, our results demonstrate a novel interplay between the gut microbiome and metabolome.

## Introduction

Many modern human life activities, such as nocturnal social activities, shift work, and jet lag (JL), can cause circadian rhythm disturbance and thus increase the risk of developing cardiovascular diseases, metabolic syndromes, and cancer, as a result of influencing the circadian clock (Rijo-Ferreira and Takahashi, 2019). Moreover, a large population in the world is suffering from sleep disorders, which may lead to circadian misalignment (Potter et al., 2016). Night shift is associated with more severe gastrointestinal (GI) symptoms (Caruso et al., 2004). Circadian rhythm disturbance can also impair intestinal barrier function and aggravate alcohol-induced hepatic pathology and inflammation (Summa et al., 2013; Gao et al., 2019).

The intestinal microorganisms are the second genome of the human body with a number that is 10 times larger than that of total somatic and germ cells of mammals (Hooper and Macpherson, 2010). The homeostasis of intestinal microbiota is essential for GI function and human health. They can also interact with the brain *via* gut microbiota-brain axis (Mayer et al., 2015). It has been demonstrated that host circadian disruption can result in the dysbiosis of the gut (such as the large intestine) microbiota (Thaiss et al., 2014; Gao et al., 2019). Small intestinal microbes also have essential physiological effects on the host (Tuganbaev et al., 2020). Research has shown that the jejunum, as the middle third of the small intestine, contains a distinctive bacterial population that differs from that in the colon, which plays a major role in absorption of carbohydrates, amino acids, small peptides, and vitamins from nutritional sources (Sundin et al., 2017). However, little is known about the effect of circadian disruption on jejunal microorganisms.

Gut microbes are regarded as a metabolic “organ” or a virtual endocrine organ (Evans et al., 2013). Indeed, bioactive molecules produced or transformed by gut microorganisms interact with the host physiology and trigger responses locally and remotely (Michaudel and Sokol, 2020). It was demonstrated that gut microbiota had their own diurnal oscillations in composition and function, and the microbes and their metabolites might be associated with the detrimental consequences of circadian disturbance for host metabolism (Thaiss et al., 2014; Alvarez et al., 2020). Microbiota-generated metabolites bridge the host-microbiota interactions and may be essential for human physiology. They may also affect the susceptibility of the host to obesity, diabetes, and immune-mediated diseases (Thaiss et al., 2014; Rooks and Garrett, 2016). Currently, the role of the following three kinds of microbial metabolites in host-microbiota interactions have attracted attention of researchers: 1) short-chain fatty acids (SCFAs), produced by gut bacteria during the fermentation of dietary fibers (Tan et al., 2014); 2) bile acids, synthesized in the liver from cholesterol and transformed by intestinal microbiota into the secondary bile acids (Sayin et al., 2013); and 3) tryptophan (Try) and its derivatives, including indole, kynurenine, and downstream products [e.g., 5-hydroxytryptamine (5-HT)] (Agus et al., 2018). Several studies have demonstrated interactions between microbial metabolites and circadian rhythms (Alvarez et al., 2020; Matenchuk et al., 2020). However, currently very few studies have examined the global changes in gut microbial metabolites upon circadian rhythm disturbance.

In the present study, using a mouse model of long-term (4 months) JL (Thaiss et al., 2014), we investigated the influence of circadian disruption on intestinal microbiome of both the middle (the jejunum) and lower (the colon) digestive tract. We also observed the changes in microbial metabolites in jet-lagged mice and explored the correlation of gut microbiota and metabolomics.

## Materials and Methods

### Animals

Male C57BL/6J mice (8–10 weeks old) were purchased from Shanghai Laboratory Animal Center, Chinese Academy of Science (Shanghai, China). The animals were housed in a constant temperature room (22–24°C) and allowed to acclimatize to the animal facility environment for 2 weeks before the experimentation, with food and water ad libitum. In the normal rhythm group, mice were kept under strict 12 h light/12 h dark cycles with lights being turned on at 6 a.m. and turned off at 6 p.m. In the JL group, jet lag was induced according to the procedure described in earlier studies (Thaiss et al., 2014), in which mice were subjected to daily 8-h advance of the light/dark cycle for every 3 days. Briefly, mice were shifted between normal light conditions (lights on at 6 a.m. and off at 6 p.m.) and an 8-h time in advance (lights on at 10 p.m. and off at 10 a.m.) every 3 days. During the process of modelling, both control mice and jet-lagged mice were fed with a standard chow diet (Purina Laboratory Rodent Diet #5001). After 4 months, the stool samples and jejunal contents of mice were collected into sterile 1.5 mL Eppendorf microcentrifuge tubes (Corning, NY, USA). The collection of mouse jejunal contents was conducted in the clean bench. The jejunum was cut open with a sterile ophthalmic scissor and the contents were collected gently with sterile tweezers. The fresh pellets and jejunal contents were immediately frozen in liquid nitrogen and stored at -80°C. All experimental protocols conformed to the National Institutes of Health (NIH) Guidelines for the Care and Use of Laboratory Animals and were approved by the School of Medicine, Shanghai Jiao Tong University.

### 16S rRNA Gene Sequencing and Data Analysis

DNA was extracted from the samples using a QIAamp Fast DNA Stool Mini Kit (Qiagen, CA, USA) according to Manufacturer’s instructions. The concentration of bacterial DNA was measured using a NanoDrop 2000 spectrophotometer (Thermo Scientific, USA). Afterwards, the 16S rRNA genes were amplified with the bacterial primer pair 338F-806R flanking the V3–V4 region using FastPfu Polymerase. Amplicons were then purified by gel extraction (AxyPrep DNA GelExtraction Kit, Axygen Biosciences, CA, USA) and quantified using QuantiFluor-ST (Promega, Madison, WI, USA). Paired-end sequencing was performed using an Illumina MiSeq system (Illumina, CA, USA). High-throughput pyrosequencing of PCR products was then performed on the free online platform of Majorbio Cloud Platform (www.majorbio.com).

Sequencing reads were demultiplexed and filtered. Operational taxonomic units (OTUs) were picked at 97% similarity cutoff, and the identified taxonomy was then aligned using the Greengenes database (Version 13.8). The relative species abundance in each group was evaluated based on the rank-abundance curves, and alpha-diversity index (Shannon and Simpson) was analyzed. For beta-diversity analysis, principal component analysis (PCA), principal coordinate analysis (PCoA), and nonmetric multidimensional scaling (NMDS) were performed using Quantitative Insights into Microbial Ecology (QIIME) 1.9.1. Difference between groups was tested using analysis of similarities (ANOSIM). For the analysis of differences in bacterial composition, we used student *t* test with *P* < 0.05 as a threshold. The calculated *P* value underwent False Discovery Rate (FDR) correction and FDR *P* < 0.05 was considered statistically significant. From phylum to genus, the biomarkers in the two groups were quantitatively analyzed by the linear discriminant analysis (LDA) effect size (LEfSe) analysis. The LEfSe analysis, with the LDA threshold of >2, was performed using the non-parametric Kruskal-Wallis (KW) test to recognize the most differently abundant taxa.

### Sample Preparation for Targeted Metabolomics Profiling

Targeted metabolomics analysis of fecal samples was performed by Metabo-Profile Biotechnology (Shanghai, China), according to the methods described previously (Choi et al., 2020; Xie et al., 2021). Briefly, the fecal samples were thawed on ice. About 5 mg of each sample was added to 25 μL of ultrapure water and homogenized with zirconium oxide beads for 3 min before addition of methanol (120 μL) containing the internal standard to extract the metabolites. Then the samples were homogenized for another 3 min and centrifuged at 18,000*g* for 20 min. The supernatants (20 μL for each sample) were transferred to a 96-well plate. The following procedures were performed on an Eppendorf epMotion Workstation (Eppendorf Inc., Hamburg, Germany). The freshly prepared derivative reagents (20 μL) were added to each well. The plate was sealed, and the derivatization was carried out at 30°C for 60 min. After derivatization, 330 μL of ice-cold 50% methanol solution was added to dilute the samples. Then, the plate was stored at –20°C for 20 min, followed by centrifugation (at 4000*g*) at 4°C for 30 min. Thereafter, 135 μL of the supernatant for each sample was transferred to a new 96-well plate with 10 μL of internal standards in each well. Serial dilutions of derivatized stock standards were added to the wells on the left. Finally, the plate was sealed for analysis. All the standards were obtained from Sigma–Aldrich (MO, USA).

### UPLC-MS/MS

An ultra-performance liquid chromatography coupled to tandem mass spectrometry (UPLC-MS/MS) system (ACQUITY UPLC-Xevo TQ-S, Waters Corp., MA, USA) was used to analyze 11 targeted metabolites of interest. We used UPLC columns including an ACQUITY HPLC BEH C18 1.7 µm VanGuard pre-column (2.1 × 5 mm) and an ACQUITY HPLC BEH C18 1.7 µm analytical column (2.1 × 100 mm) to perform chromatographic separation of fecal samples at a constant temperature of 40°C. The injection volume of sample was 5 µL. The mobile phases: eluent A was 0.1% formic acid in water, and eluent B was acetonitrile/IPA (70:30). The gradient elution condition was: 0–1 min (5% B), 1–11 min (5–78% B), 11–13.5 min (78–95% B), 13.5–14 min (95–100% B), 14–16 min (100% B), 16–16.1 min (100-5% B), 16.1–18 min (5% B). The flow rate was set at 0.40 mL/min. For mass spectrometer, capillary: 1.5 (ESI+), 2.0 (ESI-) Kv; source temperature: 150°C; desolvation temperature: 550°C; desolvation gas flow: 1000 L/h. The quality control samples were prepared along with the test samples and run after each 14 samples to ensure reproducibility.

For data analysis, the raw data files generated by UPLC-MS/MS were processed with MassLynx software (Version 4.1, Waters, MA, USA) to perform peak integration, calibration, and quantitation for each metabolite. The powerful package R studio was used for statistical analysis. PCA, partial least square discriminant analysis (PLS-DA), and orthogonal partial least square discriminant analysis (OPLS-DA) were used for classification and identification of differently altered metabolites. Potential biomarkers of differential metabolites were characterized by variables with a variable influence on projection (VIP) > 1 and *P* < 0.05 in the Student *t* test or Wilcoxon test. Z-transform was conducted to observe the distribution of different metabolites between groups. The pathway enrichment analysis was performed using pathway impact and hypergeometric test. Also, Spearman’s rank correlation analyses were conducted to explore the correlation between metabolites and gut microbiota.

## Results

### Effect of JL on Colonic Microbiota

**Figure 1:**
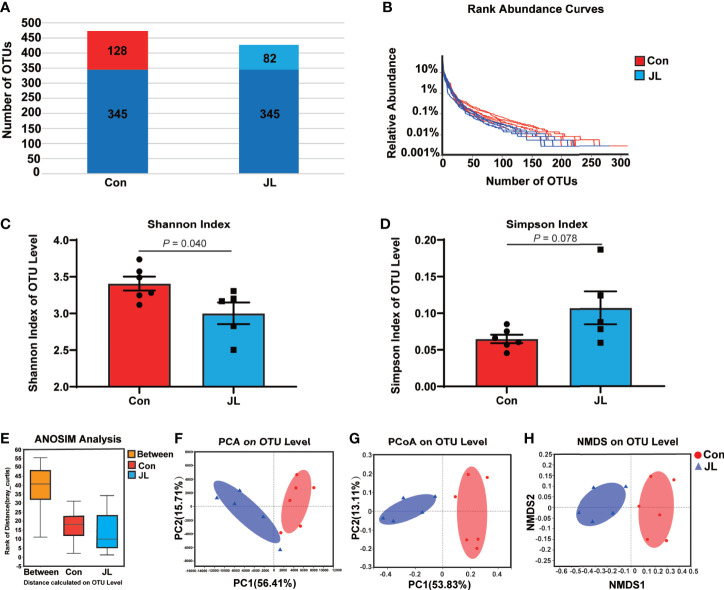
Comparison of bacterial components, abundance, α-diversity (Shannon index and Simpson index), ANOSIM analysis, and β-diversity (PCA, PCoA, and NMDS) based on OTU level in colonic microbiota of normal rhythm mice (Con; n = 6) and jet-lagged mice (JL; n = 5). **(A)** The number of OTUs in Con and JL groups. The identical OTUs in two groups were marked in dark blue. **(B)** Rank-abundance curves indicate the abundance of colonic microbiota of two groups. **(C)** Shannon index, and **(D)** Simpson index of Con and JL groups. **(E)** ANOSIM analysis between two groups. **(F)** PCA, **(G)** PCoA, and **(H)** NMDS analysis of colonic microbiota. Values are presented as the means ± SEM. α-diversity was assessed by student’s t test and β-diversity was assessed by ANOSIM analysis.

**Figure 2:**
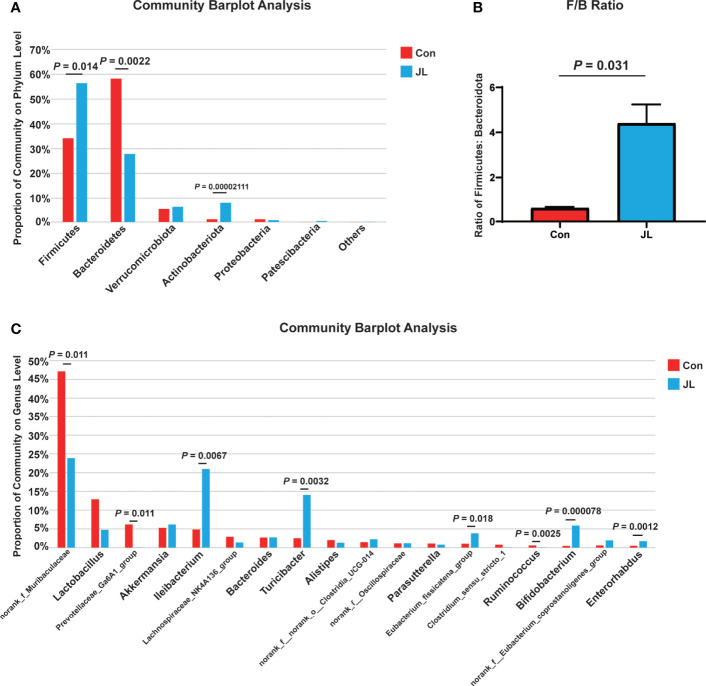
The differences of colonic microbiota between Con (n = 6) and JL (n = 5) groups. **(A)** Community barplot on phylum level of each group. **(B)** Ratio of Firmicutes to Bacteroidota. **(C)** Community barplot on genus level (top 18) of Con and JL groups. Differences were assessed by student’s t test.

**Figure 3:**
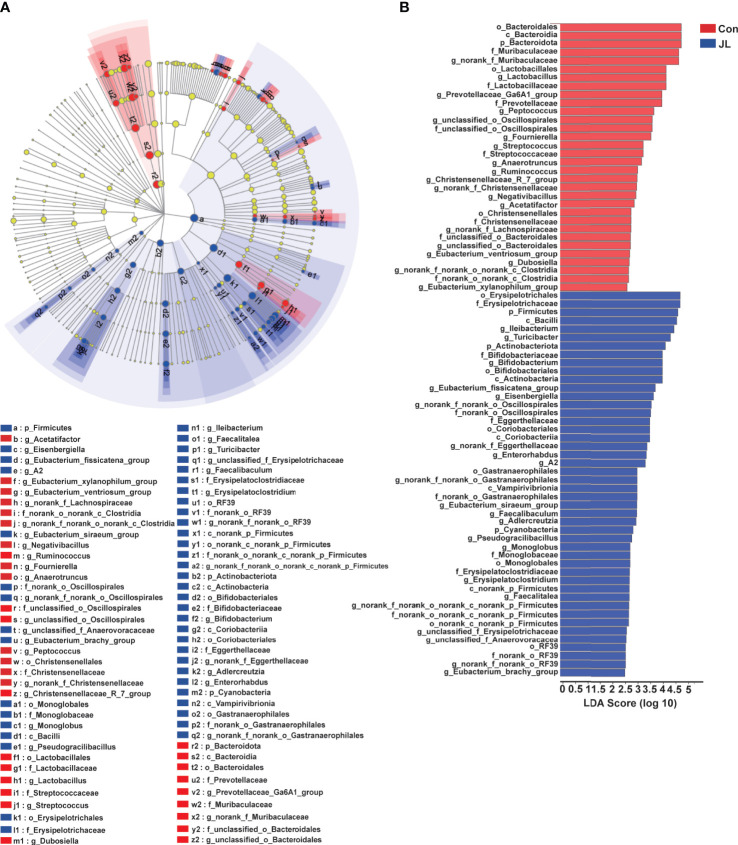
LEfSe analysis of colonic microbiota in Con and JL groups. **(A)** Cladogram indicating the enriched taxa of colonic microbiome in Con (n = 6) and JL (n = 5) groups. The central point represents the root of the tree (Bacteria), and each ring represents the next lower taxonomic level (phylum to genus: p, phylum; c, class; o, order; f, family; g, genus). The diameter of each circle represents the relative abundance of the taxon. The different taxon was assessed by non-parametric factorial Kruskal-Wallis (KW) sum-rank test. **(B)** Taxa with a different abundance in the colonic microbiota between Con and JL groups are indicated. A cut‐off value ≥ 2.0 was used for the linear discriminant analysis (LDA).

We used 16S rRNA high-throughput sequencing to determine whether circadian disruption influenced colonic microbiota. Sequencing analysis of fecal microbe revealed 551,482 raw reads. Based on the 97% similarity level, all the effective reads were clustered into operational taxonomic units (OTUs). According to the histogram of species analysis on OTU level (**Figure 1A**), the Con and JL groups had 345 identical OTUs. In addition, 128 OTUs exclusively belonged to the Con group, while only 82 OTUs belonged to the JL group. Rank-Abundance curves (**Figure 1B**) showed that the relative abundance of colonic microbiota was decreased in the JL group. Moreover, as shown in **Figures 1C, D** on α-diversity analysis, the community diversity of the colonic microbiota was decreased in JL group. Quantitative analysis showed that Shannon index was decreased by 11.9% (*P* = 0.040). Simpson index showed a tendency to increase (by 65.1%, *P* = 0.078). As shown in **Figure 1E**, the results of ANOSIM indicated that the colonic microbial structure was significantly different between the two groups (R = 0.813 and *P* = 0.003). β-diversity analysis (PCA, PCoA, NMDS) revealed a distinct clustering of colonic microbiota in the two groups (ANOSIM analysis; P = 0.003; **Figures 1F–H**). Next, the phylum-level analysis indicated that the relative abundance of Bacteroidetes phylum was significantly decreased (*P* = 0.002, FDR *P* = 0.016). The abundance of Actinobacteria was increased in the JL group (*P* = 0.00002, FDR *P* = 0.00032) and that of Firmicutes showed a tendency to increase (*P* = 0.014, FDR *P* = 0.070) for FDR *P* were greater than 0.05 (**Figure 2A**). As shown in **Figure 2B**, the ratio of Firmicutes to Bacteroidetes (F:B) was increased in the JL group (*P* = 0.031). On genus level, we chose the top 18 abundance genus for analysis. The relative abundance of Bifidobacterium (*P* = 0.000078, FDR *P* = 0.0054) was significantly increased in the JL group. The abundance of Turicibacter (*P* = 0.003, FDR *P* = 0.086), Ileibacterium (*P* = 0.0067, FDR *P* = 0.15) and Eubacterium_fissicatena_group (*P* = 0.018, FDR *P* = 0.18) showed a tendency to increase. The abundance of norank_f_Muribaculaceae (*P* = 0.011, FDR *P* = 0.16) and Prevotellaceae_Ga6A1_group (*P* = 0.011, FDR *P* = 0.16) showed a tendency to decrease in the JL group (**Figure 2C**). These results suggested that JL suppressed the abundance, richness, and diversity of colonic microbiota but increased the F: B ratio. Finally, LEfSe analysis was performed to identify specific bacteria associated with JL. The results showed that Erysipelotrichales, Erysipelotrichaceae, Bacilli, Ileibacterium, Turicibacter, Actinobacteriota, and Bifidobacteriaceae dominated in the JL group (**Figures 3A, B**).

### Effect of JL on Jejunal Microbiota

**Figure 4:**
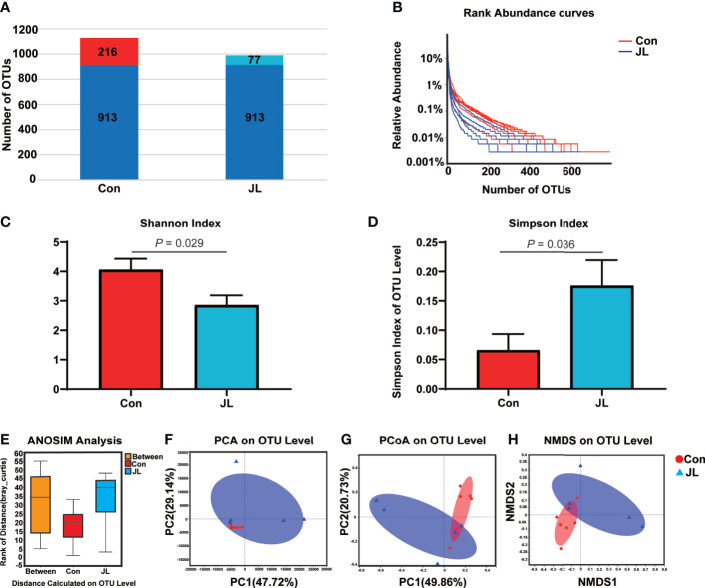
Comparison of bacterial components, abundance, α-diversity (Shannon index and Simpson index), ANOSIM analysis, and β-diversity (PCA, PCoA, and NMDS) based on OTU level in jejunal microbiota of normal rhythm mice (Con; n = 6) and jet-lagged mice (JL; n = 5). **(A)** Histogram shows the number of OTUs in the Con and JL group. **(B)** Rank-abundance curves. **(C)** Shannon index and **(D)** Simpson index of Con and JL groups. **(E)** ANOSIM analysis between two groups. **(F)** PCA, **(G)** PCoA, and **(H)** NMDS analysis of jejunal microbiota. Values are presented as the means ± SEM. α-diversity was assessed by student’s t test and β-diversity was assessed by ANOSIM analysis.

**Figure 5:**
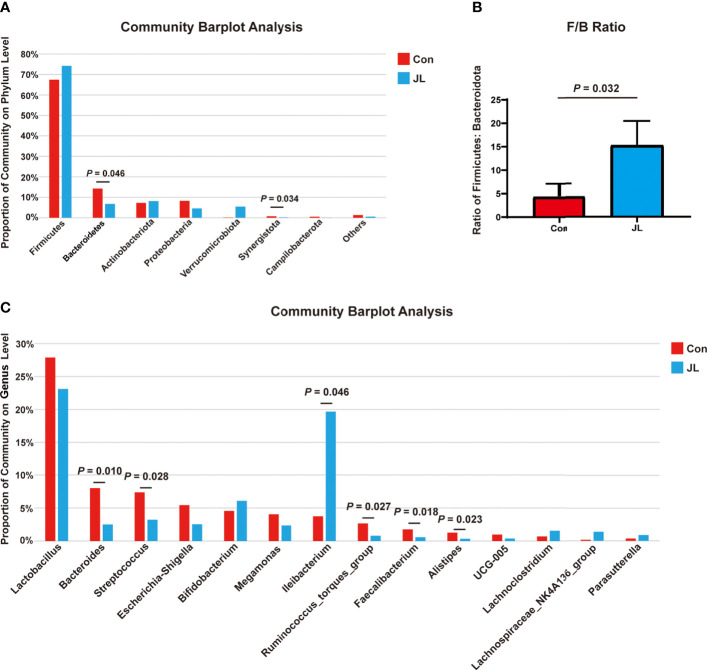
The differences of jejunal microbiota between Con (n = 6) and JL (n = 5) groups. **(A)** Community barplot on phylum level of each group in jejunum. **(B)** Ratio of Firmicutes to Bacteroidota. **(C)** Proportion of top 15 bacteria on genus level of Con and JL groups. Differences were assessed by student’s t test.

**Figure 6:**
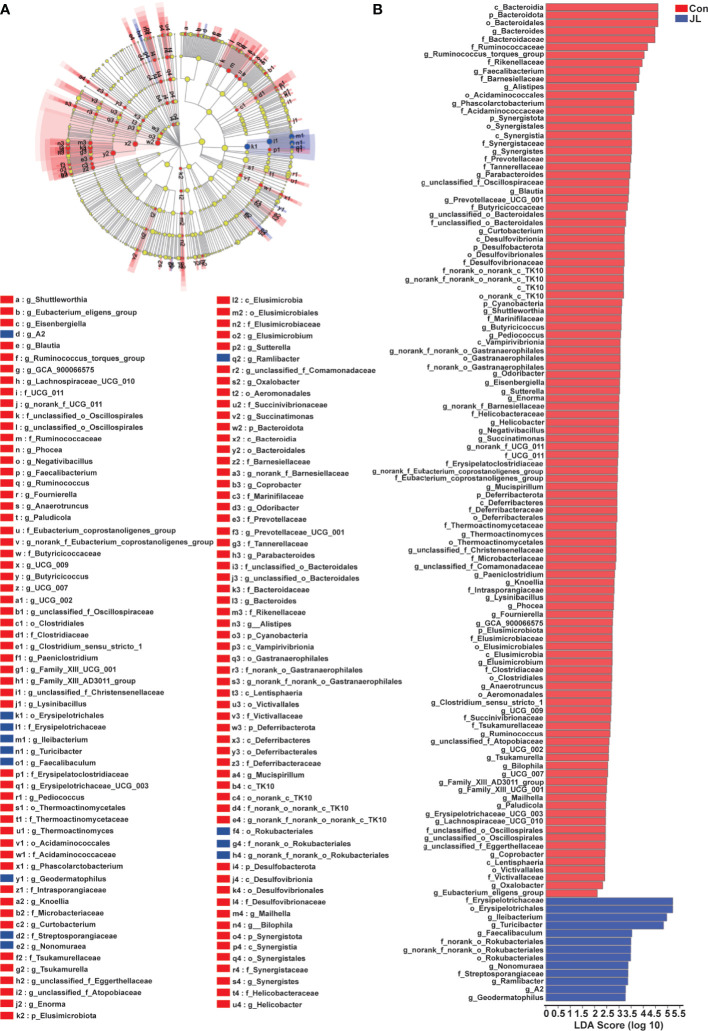
LEfSe analysis of jejunal microbiota in Con (n = 6) and JL (n = 5) groups. **(A)** Taxonomic cladogram obtained from LEfSe analysis. The different taxon was assessed by non-parametric factorial Kruskal-Wallis (KW) sum-rank test. Different taxa are highlighted by colored circles and shaded areas. The diameter of each circle reflects the abundance of that taxa in the community. **(B)** Taxa with a different abundance in the jejunal microbiota between Con or JL groups. A cut‐off value ≥ 2.0 was used for the linear discriminant analysis (LDA).

The results of 16S rRNA high-throughput sequencing showed that jejunal microbiota might also be affected by JL. Sequencing analysis revealed 569,943 raw reads. According to a 97% similarity level, all effective reads were clustered into OTUs. **Figure 4A** shows species distribution in the Con and JL groups on OTU level. The two groups have 913 identical OTUs. Further, 216 OTUs exclusively belonged to the Con group, while 77 OTUs belonged to JL group. Rank-abundance curves revealed that the richness of the jejunal microbiota was decreased by JL (**Figure 4B**). In addition, α-diversity analysis showed an overt difference in jejunal microbiota between the Con and JL groups. Shannon index was significantly decreased by 29.6% (*P* = 0.029) (**Figure 4C**), while Simpson index was markedly increased by 167.7% (*P* = 0.036) in the JL group (**Figure 4D**). As is shown in **Figure 4E**, the results of ANOSIM analysis revealed a significant difference between two groups (R = 0.2773, *P* = 0.033). β-diversity analysis showed an obvious clustering of jejunal microbiota composition between Con and JL groups using PCoA and NMDS analyses (**Figures 4G, H**), while no obvious separation of the two groups was observed using PCA analysis (**Figure 4F**). These results suggested that JL could affect the abundance, richness, and diversity of jejunal microbiome. According to the community analysis, JL tended to decrease the relative abundance of Bacteroidota phylum (*P* = 0.046, FDR *P* = 0.18) in the jejunal microbiota. The abundance of Firmicutes showed a tendency to increase in the JL group, with no statistically significant difference (**Figure 5A**). Chronic JL caused an increase in the ratio F: B (*P* = 0.032) (**Figure 5B**). Further, on the genus level, **Figure 5C** showed that the abundance of Ileibacterium (*P* = 0.046, FDR *P* = 0.34) showed a tendency to increase in the JL group, while the abundance of Streptococcus (*P* = 0.028, FDR *P* = 0.34), Bacteroides (*P* = 0.010, FDR *P* = 0.34), Ruminococcus_torques_group (*P* = 0.027, FDR *P* = 0.34), and Faecalibacterium (*P* = 0.018, FDR *P* = 0.34) showed a tendency to decrease after JL. All the *P* values were greater than 0.05 after FDR correction, indicating the changes of jejunal microbiota caused by chronic JL may be relative slight compared with colonic microbiota. Furthermore, LEfSe analysis was performed to recognize the specific microbiota related to JL (**Figures 6A, B**). Five types of bacteria dominated in JL group, including Erysipelotrichaceae, Erysipelotrichales, Ileibacterium, Turicibacter, and Faecalibaculum. These results indicated that, besides colonic microbiota, the bacterial community of jejunum might also be affected by chronic JL treatment.

### Influence of JL on Fecal Metabolites

**Figure 7:**
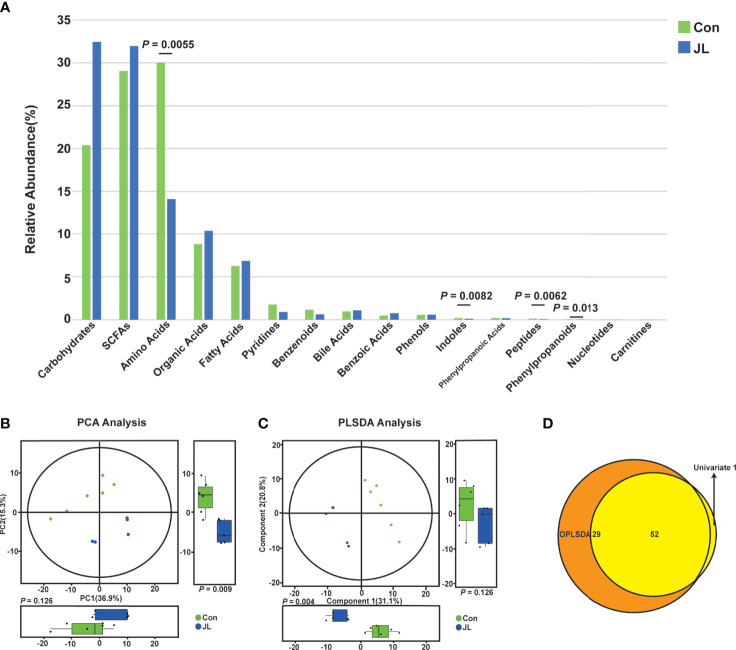
The difference of fecal metabolites between Con (n = 6) and JL (n = 5) groups. **(A)** Histogram showing abundance of different kinds of fecal metabolites in two groups. Differences between two groups were assessed by student’s t test or Wilcoxon test. **(B)** PCA and difference analysis on both dimensions. **(C)** PLS-DA analysis on both dimensions. **(D)** Venn diagrams of differential metabolites screened by multi-dimensional (PLS-DA) and single-dimensional. Single-dimensional was performed by student’s t test or Wilcoxon test.

The fecal metabolome represents a functional readout of the activity of gut microbiome, and thus can serve as an intermediary phenotype reflecting the host-microbiome interactions (Liu et al., 2020). We subsequently analyzed the fecal metabolites of Con and JL mice using a targeted UPLC-MS/MS based metabolomics approach. As shown in **Figure 7A**, the relative abundance of fecal metabolites in JL mice was different from that in the Con group. The abundance of amino acids (14.11% *vs* 30.04%, *P* = 0.006), indoles (0.11% *vs* 0.20%, *P* = 0.008), peptides (0.06% *vs* 0.11%, *P* = 0.006) and phenylpropanoids (0.03% *vs* 0.05%, *P* = 0.013) in fecal metabolites were decreased in the JL group compared with the Con group. The abundance of carbohydrates and SCFAs was similar between two groups.

**Figure 8:**
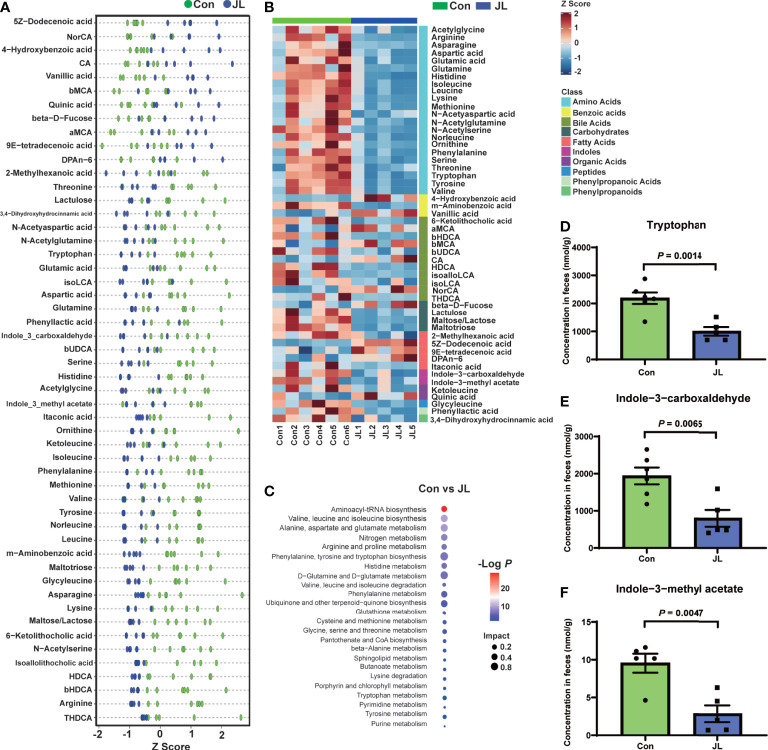
Potential biomarkers of Con (n = 6) and JL (n = 5) groups. **(A)** Scatter plot of Z scores in each group. **(B)** Heatmap of Z scores in each group. Blocks in red and blue denote high and low z-score values of potential biomarkers, respectively. **(C)** Bubble chart of pathway analysis of differential metabolites. **(D–F)** The concentrations of tryptophan and its derivatives in Con and JL group. **(D)** Tryptophan, **(E)** Indole-3-carboxaldehyde, **(F)** Indole-3-Methyl acetate. Differences were assessed by student’s t test.

In addition, the results of PCA and PLS-DA analysis showed apparently distinct clustering of fecal metabolites (**Figures 7B, C**). Based on the results of both univariate and multivariate statistical analyses for the differential metabolites, 52 potential biomarkers which have biological significance were obtained (**Figure 7D**). A scatter plot and a heatmap of Z scores were obtained after the Z-transform of each biomarker (**Figures 8A, B**). Based on the above-mentioned results and pathway clustering analysis shown in **Figure 8C**, we subsequently explored Try and its derivatives, bile acids, and itaconic acids. Among them, Try had the largest impact value. Compared with the control group, the levels of Try (1001.46 ± 343.55 *vs* 2185.52 ± 502.33 nmol/g, *P* = 0.0014), indole-3-carboxaldehyde (IAId; 802.04 ± 509.31 *vs* 1937.13 ± 553.35 nmol/g, *P* = 0.0065), and indole-3-methyl acetate (2.86 ± 2.49 *vs* 7.98 ± 4.59 nmol/g, *P* = 0.047) were all significantly decreased in the JL group (**Figures 8D–F**). Bile acids are also important bacterial metabolites in the intestine. Via gut microbiome–mediated metabolism, the primary bile acids are transformed into the secondary bile acids, namely deoxycholic acid (DCA), lithocholic acid (LCA) and ursodeoxycholic acid (UDCA) (Bajor et al., 2010; Jia et al., 2018). The results showed that compared with Con group, the levels of the secondary bile acids and their hepatic microsomal metabolites, such as isoalloLCA (3.58 ± 1.54 *vs* 22.19 ± 14.66 nmol/g, *P* = 0.030), β-UDCA (23.34 ± 13.22 *vs* 59.05 ± 24.44 nmol/g, *P* = 0.045), isoLCA (10.57 ± 8.09 *vs* 23.88 ± 11.03 nmol/g, *P* = 0.047), 6-ketolithocholic acid (59.40 ± 47.09 *vs* 316.31 ± 142.46 nmol/g, *P* = 0.0043), hyodeoxycholic acid (HDCA; 224.18 ± 98.07 *vs* 1594.63 ± 870.24 nmol/g, *P* = 0.0043), β-HDCA (38.45 ± 15.97 *vs* 276.91 ± 145.94 nmol/g, *P* = 0.0043), and taurine-hyodeoxycholic acid (THDCA; 2.27 ± 1.78 *vs* 33.83 ± 37.83 nmol/g, *P* = 0.0043) were decreased in the jet-lagged mice (**Figures S1A–G**). Whereas, the levels of other secondary bile acids, such as α-muricholic acids (αMCA; 953.74 ± 298.75 *vs* 494.93 ± 340.21 nmol/g, *P* = 0.041) and βMCA (828.47 ± 336.09 *vs* 382.51 ± 269.34 nmol/g, *P* = 0.045), as well as the levels of the primary bile acids such as cholic acid (CA; 585.34 ± 317.84 *vs* 181.05 ± 132.39 nmol/g, *P* = 0.044) and norcholic acid (NorCA; 39.52 ± 16.41 *vs* 9.32 ± 12.48 nmol/g, *P* = 0.017), were increased in the jet-lagged mice (**Figures S1H–K**). The level of itaconic acid, which is a major physiological regulator of the global metabolic rewiring, was decreased in JL group (2.79 ± 0.63 *vs* 8.28 ± 5.37 nmol/g, *P* = 0.0043) (**Figure S2**). Therefore, chronic JL could not only change the microbiome of both jejunum and colon but also disturb the concentration of fecal metabolites.

### Correlation Analysis of Colonic Microbiota and Fecal Metabolites

**Figure 9:**
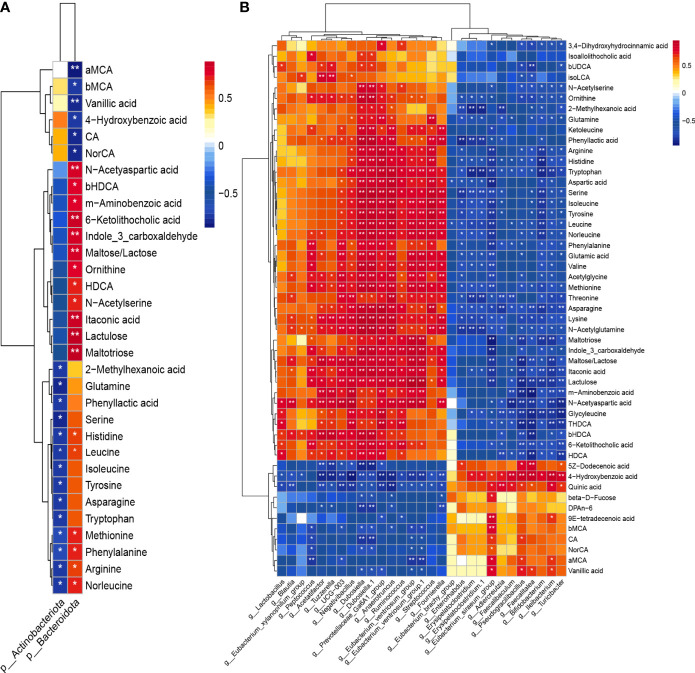
Heatmap of Spearman’s correlation coefficients between colonic microbiota and fecal metabolites on phylum level **(A)** and on genus level **(B)**. Blocks in red and blue denote high and low correlation coefficients, respectively. *P < 0.05, **P < 0.01.

We subsequently analyzed the correlation between colonic microbiota and its metabolites (**Figures 9A, B**). On phylum level, the abundance of Try was negatively correlated with that of *Actinobacteriota* (r = -0.76, *P* = 0.016). The abundance of IAId, a downstream product of Try, was positively correlated with that of *Bacteroidetes* (r = 0.82, *P* = 0.0068). On the genus level, Try level was positively correlated with abundance of *Prevotellacea UCG-003* (r = 0.68, *P* = 0.030), *Negativibacillus* (r = 0.78, *P* = 0.0084), *Dubosiella* (r = 0.81, *P* = 0.0041), *Prevotellaceae_Ga6A1_group* (r = 0.89, *P* = 0.00065), *Anaerotruncus* (r = 0.87, *P* = 0.00095), *Ruminococcus* (r = 0.76, *P* = 0.016), *Eubacterium_ventriosum_group* (r = 0.77, *P* = 0.0096), *Streptococcus* (r = 0.77, *P* = 0.0099), and *Fournierella* (r = 0.75, *P* = 0.013). On the contrary, it was negatively correlated with the abundance of bacteria including *Enterorhabdus* (r = -0.75, *P* = 0.018), *Erysipelatoclostridium* (r = -0.85, *P* = 0.0035), *Eubacterium_siraeum_group* (r = -0.81, *P* = 0.0041), *Adlercreutzia* (r = -0.68, P = 0.030), *Faecalibaculum* (r = -0.70, P = 0.026), *Pseudogracilibacillus* (r = -0.71, *P* = 0.022), *Faecalitalea* (r = -0.69, *P* = 0.028), *Bifidobacterium* (r = -0.85, *P* = 0.0035), *Ileibacterium* (r = -0.81, *P* = 0.0082), and *Turicibacter* (r = -0.75, *P* = 0.012). The level of IAId was positively correlated with the abundance of *Peptococcus* (r = 0.75, *P* = 0.012), *Acetatifactor* (r = 0.66, *P* = 0.036), *Prevotellacea UCG-003* (r = 0.74, *P* = 0.014), *Negativibacillus* (r = 0.70, *P* = 0.026), *Dubosiella* (r = 0.86, *P* = 0.0015), *Prevotellaceae_Ga6A1_group* (r = 0.78, *P* = 0.0075), *Anaerotruncus* (r = 0.83, *P* = 0.0028), *Ruminococcus* (r = 0.82, *P* = 0.0068), *Eubacterium_ventriosum_group* (r = 0.88, *P* = 0.00085), *Streptococcus* (r = 0.72, *P* = 0.020), and *Fournierella* (r = 0.76, *P* = 0.01). However, it was negatively correlated with the abundance of *Eubacterium_siraeum_group* (r = -0.87, *P* = 0.0011), *Pseudogracilibacillus* (r = -0.71, *P* = 0.022), *Bifidobacterium* (r = -0.72, *P* = 0.024), *Ileibacterium* (r = -0.68, *P* = 0.035), and *Turicibacter* (r = -0.64, *P* = 0.047).

## Discussion

The gut microbiota and its metabolites play a key role in mammalian physiology. Gut microbiome can be disturbed by a variety of factors, such as environment, diet, and medications. The present study showed that JL-induced circadian disruption might exacerbate jejunal and colonic microenvironment *via* influencing gut microbes and their metabolism. Our results, in line with those of previous studies (Thaiss et al., 2014; Gao et al., 2019), suggested that gut microbial dysbiosis, which may lead to metabolic imbalance, might be an important mechanism in circadian disturbance-related human diseases.

As for colonic microbiome, we found that, similar to the results of the previous studies (Thaiss et al., 2014), chronic jet lag caused dysbiosis of microbial community. In the present study, 16s rRNA sequencing revealed that the abundance, richness, and diversity of colonic microbiota were decreased in the jet-lagged mice. Moreover, induction of jet lag increased the F:B ratio, an indicator for the imbalance of gut microbiota (De Luca and Shoenfeld, 2019; Crovesy et al., 2020; Grigor’eva, 2020; Stojanov et al., 2020). Studies have shown that obese animals and humans had a higher F:B ratio compared with normal weight individuals (Magne et al., 2020). It should be noted that examining the F:B ratio is only one way to assess changes in the microbiome as a whole and other factors need to be considered. We also found that the abundance of *Actinobacteria* phylum was increased in jet-lagged mice. Moreover, the abundance of *Bifidobacterium* genus, which belongs to *Actinobacteria* phylum was also increased after JL. It has been reported that the members of this genus are regarded as probiotic demonstrating therapeutic effects in many GI diseases (Hidalgo-Cantabrana et al., 2017; Cukrowska et al., 2020). This is contradictory to our findings showing that JL worsened the intestinal microenvironment. Therefore, it was supposed that *Actinobacteria* and *Bifidobacterium* might play a minor role in correcting the perturbation of gut microbiome in jet-lagged mice because they only held a small percentage of the whole gut bacteria (Binda et al., 2018). However, further work is required to confirm this hypothesis. On genus level, another bacterium that attracted attention is *Turicibacter*. Studies have shown that *Turicibacter* might belong to a hypothetical pro-inflammatory group, and ketogenic diet can reduce the relative abundance of *Turicibacter* (Ma et al., 2018). Our results showed that the abundance of the bacterium *Turicibacter* was increased in jet-lagged mice, indicating JL might produce potential pro-inflammatory effect.

The effect of circadian disruption on jejunal microorganisms is not clear. We therefore explored whether JL influences the jejunal microbiota. Similar to the results of colonic microbiome, the abundance, richness, and diversity of jejunal microbiota were also decreased in the jet-lagged mice. However, it seemed that chronic JL had a slight impact on the jejunal microbiome based on FDR adjusted *P* values. The F:B ratio was also increased in jejunum in jet-lagged mice, indicating the dysbiosis of gut microbiota in the middle GI tract after JL treatment.

Since the jet-lagged mice demonstrated disturbed homeostasis of gut microbiota, we hypothesized alterations in microbial metabolism might be at least partially affected by abnormal rhythm. Therefore, we subsequently performed targeted UPLC-MS/MS based metabolomics analysis of fecal samples to examine whether circadian rhythm disturbance affects microbial metabolites. The analysis results revealed similar abundance of carbohydrates and SCFAs between Con and JL groups. Notably, the fecal concentration of amino acids was decreased in JL group. Among various amino acids, Try is one of the essential amino acids for human body. Studies have shown that perturbations in Try metabolism may be implicated in human diseases, such as IBD, metabolic syndrome, and neuropsychiatric diseases (Agus et al., 2018). Try has been reported to alleviate colitis caused by dextran sodium sulfate (Islam et al., 2017). IAId is a microbial Try derivative. *Lactobacillus reuteri* (*L. reuteri*) and *Lactobacillus. L. johnsonii* in the intestine can produce IAId (Zelante et al., 2013; Hou et al., 2018). IAld was reported to be a direct effector of *L. reuteri* to protect the integrity of intestinal mucus and motivate epithelial hyperplasia. Therefore, Try and IAId might play a protective role in intestinal inflammation (Hou et al., 2018). We found that the fecal levels of both Try and IAId were decreased in JL group. Moreover, correlation analysis identified associations between the levels of tryptophan and its derivatives and colonic microbiota. In the present study, LEfSe analysis revealed that Lactobacillus dominates in Con group, which might partially explain our observations that the content of IAId was higher in Con group than JL group. Collectively, these results indicate that supplementation of Try and IAId might provide a protective effect on the impairment of intestinal barrier caused by chronic JL.

Primary bile acids, including chenodeoxycholic acid (CDCA) and CA, are secreted by liver into the small intestine to ensure assimilation of dietary lipids. With 95% of them actively reabsorbed in the terminal ileum, the rest are predominantly transformed by bacteria into secondary bile acids (LCA and DCA, respectively) *via* deconjugation and 7α-dehydroxylation in colon, where they are passively absorbed or excreted into feces (Bajor et al., 2010). We found that chronic JL led to obvious changes in the fecal levels of bile acids. Notably, the concentrations of several secondary bile acids and their metabolites, such as isoalloLCA [a LCA derivative that was reported to regulate host immune responses (Hang et al., 2019)], were decreased in jet-lagged mice. Studies have shown a relationship between bile acids and liver inflammation, colitis, or carcinoma (Jia et al., 2018). Therefore, it is possible that the disturbed bile acid metabolism could contribute to dysfunction and dysbiosis of the whole intestine under jet-lagged conditions. Itaconic acid plays an anti-inflammatory role during macrophage activation and liver ischemia–reperfusion injury (Lampropoulou et al., 2016). In the present study, the fecal level of itaconic acid was decreased in JL group. Therefore, the anti-inflammation mechanism could be mitigated under jet-lagged conditions as a result of reduced itaconic acid level.

Taken together, the results suggested that chronic circadian misalignment might exacerbate gut microenvironment in both jejunum and colon. The related microbes and their metabolites were analyzed in jet-lagged mice in order to provide new potential therapeutic targets for alleviating GI dysfunction of human physiology caused by aberrant circadian rhythms. However, the mouse model of circadian rhythm disorder we used might not fully simulate the characteristics of modern lifestyle changes. Therefore, our results may not completely reflect the changes caused by the disturbance of human biorhythm. In addition, the protective effect of these bacteria and their metabolites need further exploration. Another limitation is that the sample size in this study was relatively small, and a bias might exist in analyzing gut microbiome and metabolomics. Therefore, future studies with larger sample size are needed.

## Data Availability Statement

The original research showed in our study are publicly available. This data can be found on this website after release date: https://www.ncbi.nlm.nih.gov/sra/PRJNA719228, and the SRA number is SRP313173.

## Ethics Statement

All experimental protocols conformed to the Guide for the Care and Use of Laboratory Animals [National Institutes of Health (NIH), MD, USA] and were approved by the School of Medicine, Shanghai Jiao Tong University.

## Author Contributions

QL designed the research, analyzed the data and wrote the manuscript. BW, H-YQ, X-JY, LC, and Q-QW performed and participated in the experiments. S-LC obtained the funding and revised the manuscript. All the authors contributed to the article and approved the submitted version.

## Funding

This work was supported by grants from the National Natural Science Foundation of China (No. 81970473, 81670484, 81970472, 82000487, 81470812, and 81500412).

## Conflict of Interest

The authors declare that the research was conducted in the absence of any commercial or financial relationships that could be construed as a potential conflict of interest.

## Abbreviations

JL, Jet lag; IBD, inflammatory bowel disease; Try, tryptophan; IAId, indole-3-carboxaldehyde; CA, cholic acid; NorCA, norcholic acid; MCA, muricholic acids; LCA, lithocholic acid; UDCA, ursodeoxycholic acid; HDCA, hyodeoxycholic acid; OTU, operational taxonomic unit; LDA, linear discriminant analysis; LEfSe, effect size; PCA, principal component analysis, PCoA, principal coordinate analysis; NMDS, nonmetric multidimensional scaling.
