# Supplementary material for: Chronic Jet Lag Exacerbates Jejunal and Colonic Microenvironment in Mice
Source: Front Cell Infect Microbiol. 2021 Jun 1;11:648175. doi: 10.3389/fcimb.2021.648175 (PMC8204051; doi:10.3389/fcimb.2021.648175)
Supplement: Supplementary file 1 [file DataSheet_1.docx]

**Figure S1**

**
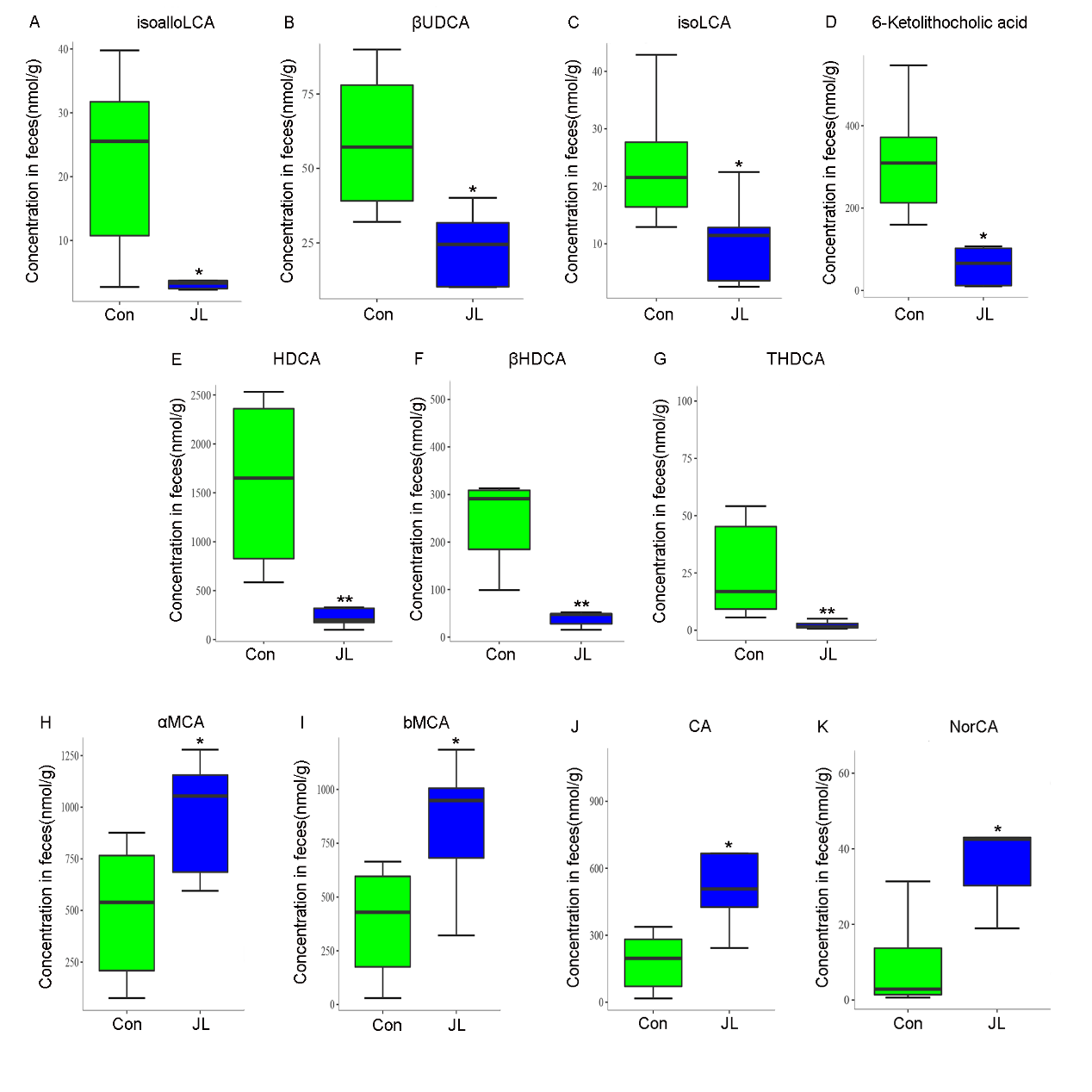
Figure S2**

**
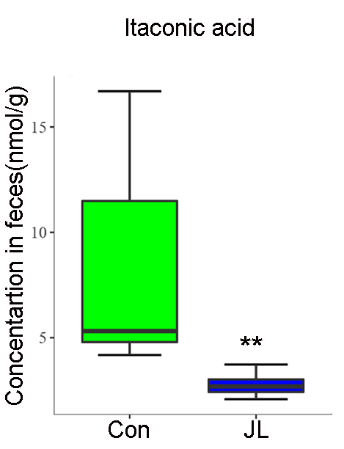
**

**Figure S1** The content of kinds of bile acids in the Con and JL group (Con: n = 6, JL: n = 5). (A) isoalloLCA, (B) β- ursodeoxycholic acid (βUDCA), (C) iso-lithocholic acid (isoLCA), (D) 6-Ketolithocholic acid, (E) hyodeoxycholic acid (HDCA), (F) β-ursodeoxycholic acid (βUDCA), (G) taurine- hyodeoxycholic acid (THDCA), (H) α-muricholic acids (αMCA), (I) β- muricholic acids (βMCA), (J) cholic acid (CA), (K) norcholic acid (NorCA). Differences were assessed by student’s t test and denoted as follows: **P* < 0.05; ***P* < 0.01.

**Figure S2** The content of itaconic acid in the Con and JL group (Con: n = 6, JL: n = 5). Differences were assessed by student’s t test and denoted as follows: ***P* < 0.01.
